# NADPH‐related processes studied with a SoxR‐based biosensor in *Escherichia coli*

**DOI:** 10.1002/mbo3.785

**Published:** 2018-12-25

**Authors:** Alina Spielmann, Meike Baumgart, Michael Bott

**Affiliations:** ^1^ IBG‐1: Biotechnology, Institute of Bio‐ and Geosciences Forschungszentrum Jülich Jülich Germany

**Keywords:** NADPH biosensor, RseC, RsxABCDGE complex, transhydrogenases PntAB and SthA

## Abstract

NADPH plays a crucial role in cellular metabolism for biosynthesis and oxidative stress responses. We previously developed the genetically encoded NADPH biosensor pSenSox based on the transcriptional regulator SoxR of *Escherichia coli*, its target promoter P*_soxS_* and eYFP as fluorescent reporter. Here, we used pSenSox to study the influence of various parameters on the sensor output in *E. coli*during reductive biotransformation of methyl acetoacetate (MAA) to (*R*)‐methyl 3‐hydroxybutyrate (MHB) by the strictly NADPH‐dependent alcohol dehydrogenase of *Lactobacillus brevis* (*Lb*Adh). Redox‐cycling drugs such as paraquat and menadione strongly activated the NADPH biosensor and mechanisms responsible for this effect are discussed. Absence of the RsxABCDGE complex and/or RseC caused an enhanced biosensor response, supporting a function as SoxR‐reducing system. Absence of the membrane‐bound transhydrogenase PntAB caused an increased biosensor response, whereas the lack of the soluble transhydrogenase SthA or of SthA and PntAB was associated with a strongly decreased response. These data support the opposing functions of PntAB in NADP^+^ reduction and of SthA in NADPH oxidation. In summary, the NADPH biosensor pSenSox proved to be a useful tool to study NADPH‐related processes in *E. coli*.

## 1 INTRODUCTION

Genetically encoded biosensors based on transcriptional regulators (TRs) and using fluorescent proteins as reporters are highly useful tools for monitoring physiological responses at the single‐cell level and for enabling high‐throughput screening in strain and enzyme development (Dietrich, McKee, & Keasling, 2010; Eggeling, Bott, & Marienhagen, 2015; Mahr & Frunzke, 2016; Rogers, Taylor, & Church, 2016). In recent years, we developed TR‐based biosensors for amino acids and used them, for example, to identify novel mutations for overproduction of l‐lysine, l‐arginine, and l‐histidine by FACS‐based screening of genome‐wide, gene‐specific, and codon‐specific mutant libraries (Binder et al., 2012; Binder, Siedler, Marienhagen, Bott, & Eggeling, 2013; Schendzielorz et al., 2014) or for increasing l‐valine production by adaptive laboratory evolution (Mahr et al., 2015).

Besides the sensors for amino acids, we also established a TR‐based biosensor responding to the intracellular availability of NADPH (Siedler et al., 2014). It is based on the SoxRS regulatory system of *Escherichia coli*, which governs the expression of oxidative stress response genes by a regulatory cascade, in which synthesis of SoxS depends on transcriptional activation of *soxS* expression by SoxR (Greenberg, Monach, Chou, Josephy, & Demple, 1990; Tsaneva & Weiss, 1990). In the genome, the *soxR* gene is located divergently to the *soxS* gene. Whereas initial studies indicated that the promoter of *soxR* is located within the *soxS* coding region (Wu & Weiss, 1991), a subsequent analysis revealed that the promoters of the two genes overlap (Hidalgo, Leautaud, & Demple, 1998). The transcription factor SoxR is a homodimer with each subunit containing an [2Fe‐2S] cluster (Hidalgo & Demple, 1994; Watanabe, Kita, Kobayashi, & Miki, 2008). SoxR activity is controlled by a change of the redox state of its [2Fe−2S] clusters, which is associated with conformational changes: only in the oxidized [2Fe−2S]^2+^ state, but not in the reduced [2Fe−2S]^+^ state, SoxR activates *soxS*expression (Ding, Hidalgo, & Demple, 1996; Gaudu & Weiss, 1996). SoxR binds to its target site, which is located between the −10 and −35 regions of the *soxS* promoter and downstream of the −10 region of the *soxR* promoter, both in the oxidized and in the reduced state with high affinity (Gaudu & Weiss, 1996; Hidalgo & Demple, 1994). Besides activating *soxS* expression in the oxidized state, SoxR simultaneously represses expression of its own gene, both in the oxidized and the reduced state (Hidalgo et al., 1998). SoxR was previously considered to activate expression of *soxS* only, but recent studies uncovered further direct SoxR target genes (Seo, Kim, Szubin, & Palsson, 2015).

SoxS functions as a transcriptional activator of genes, many but not all of which are responsible for coping with damage caused by oxygen radicals, such as *sodA* for superoxide dismutase, *zwf* for the NADPH‐generating glucose 6‐phosphate dehydrogenase, or *fumC* for fumarase C (Blanchard, Wholey, Conlon, & Pomposiello, 2007; Seo et al., 2015). It has been shown that the intrinsic instability of SoxS (*t*_1/2_ ~ 2 min) and the degradation of SoxS, primarily through the Lon protease, are responsible for the rapid return of the SoxRS system to the inactive state when the stimuli activating the system are no longer present (Griffith, Shah, & Wolf, 2004).

Current evidence indicates that there are multiple ways how the conversion of inactive, reduced SoxR into active, oxidized SoxR can be triggered. These include direct oxidation of SoxR by superoxide (Fujikawa, Kobayashi, & Kozawa, 2012; Liochev & Fridovich, 2011) and by redox‐cycling drugs (Gu & Imlay, 2011), nitrosylation of SoxR (Ding & Demple, 2000), and conditions leading to a diminished NADPH/NADP^+^ ratio within cells (Krapp, Humbert, & Carrillo, 2011; Liochev & Fridovich, 1992). The responsiveness to the NADPH availability is presumably due to the fact that SoxR is subject to permanent autoxidation under aerobic conditions, but is kept in the reduced state by NADPH‐dependent reductases (Koo et al., 2003).

In a previous study, we made use of the NADPH‐responsiveness of the SoxRS system to construct the biosensor pSenSox, in which the SoxR‐activated *soxS* promoter controls expression of the *eyfp* gene, allowing detection of SoxR activation at the single‐cell level (Siedler et al., 2014). Using the reduction of methyl acetoacetate (MAA) to (*R*)‐methyl 3‐hydroxybutyrate (MHB) by the strictly NADPH‐dependent alcohol dehydrogenase of *Lactobacillus brevis* (*Lb*Adh) as model reaction, we could show that the specific eYFP fluorescence of *E. coli*cells correlated not only with the MAA concentration added to the cells, but also with the specific *Lb*Adh activity when a fixed MAA concentration was provided. The latter property enabled high‐throughput screening of an *Lb*Adh mutant library by fluorescence‐activated cell sorting (FACS) for variants with improved activity for the alternative substrate 4‐methyl‐2‐pentanone (Siedler et al., 2014).

In this study, we employed pSenSox to test various conditions, including growth media, redox‐cycling drugs, mutants lacking SoxR reductases, and mutants lacking transhydrogenases for their influence on SoxR activity.

## 2 RESULTS AND DISCUSSION

### 2.1 Influence of different media on the NADPH biosensor response

**Figure 1:**
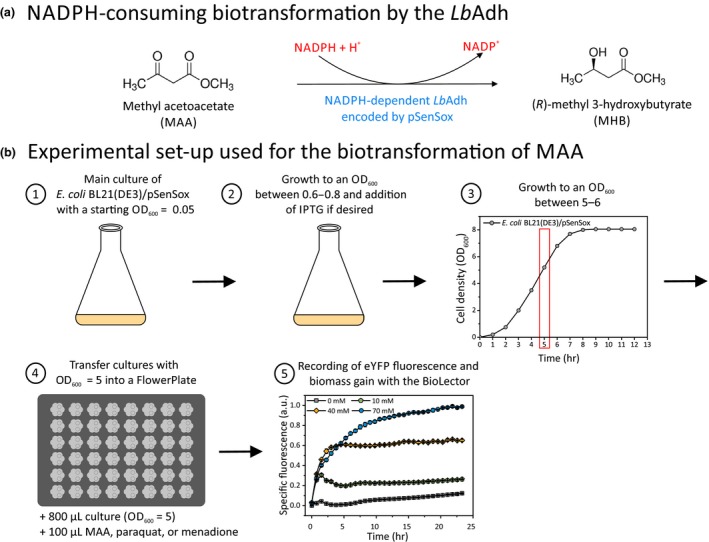
(a) NADPH‐dependent reduction of MAA to MHB by the *Lb*Adh. (b) Experimental setup used in this work to study the responses of the SoxR‐based NADPH biosensor encoded by plasmid pSenSox during whole‐cell biotransformation of MAA to MHB. (1) *Escherichia coli* BL21(DE3) carrying pSenSox was cultivated in shake flasks with a starting OD_600_ of 0.05. (2) If desired, IPTG was added to the cultures at an OD_600_ between 0.6 and 0.8. In the absence of IPTG, basal expression of the *Lbadh* gene by the *tac* promoter is sufficient for *Lb*Adh synthesis and biotransformation of MAA to MHB. However, IPTG can be added to maximize *Lbadh* expression. (3) To ensure that enough biomass is formed for the biotransformation, the cultures were grown for at least 5 hr until an OD_600_ of 5 or higher was reached. (4) For the biotransformation, 800 µl of the culture with an OD_600_ adjusted to 5 were transferred into a Flowerplate (m2p‐labs, Baesweiler, Germany) and the biotransformation was started by the addition of 100 µl MAA at the desired concentration to these cultures. To study the effect of redox‐cycling drugs, either 100 µl paraquat or 100 µl menadione were added to the cultures at the desired concentration. (5) The change in eYFP fluorescence and biomass was monitored for around 24 hr with a BioLector microcultivation system that enables online recording of eYFP fluorescence (excitation at 485 nm, emission at 520 nm) and biomass gain (change in cell density measured as backscattered light at 620 nm (Kensy, Zang, Faulhammer, Tan, & Büchs, 2009). The specific fluorescence over the time course of 24 hr corresponds to the ratio of absolute fluorescence/backscatter

**Figure 2:**
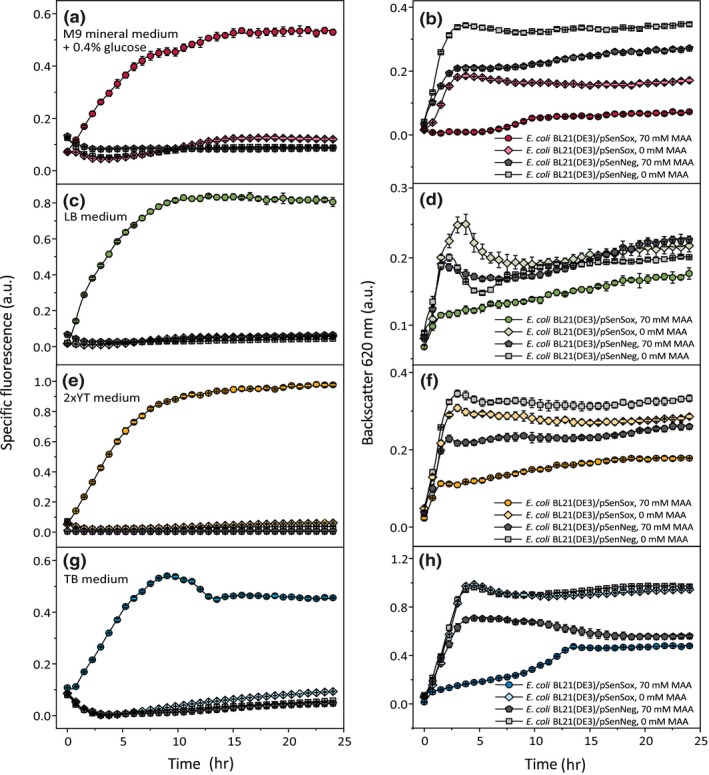
Influence of different media on the NADPH biosensor response during biotransformation of MAA to MHB using the experimental setup shown in Figure 1. The changes in fluorescence and biomass were followed during the biotransformation of MAA to MHB by the NADPH‐dependent alcohol dehydrogenase of *Lactobacillus brevis* (*Lb*Adh) using *Escherichia coli*BL21(DE3)/pSenSox and *E. coli*BL21(DE3)/pSenNeg cultures cultivated either in M9 mineral medium supplemented with 0.4% (w/v) glucose (a and b), or in LB medium (c and d), or in 2xTY medium (e and f), or in TB medium (g and h). Mean values and standard deviations of three independent biological replicates are shown. The values were normalized to the maximal backscatter value measured in TB medium and to the maximal specific fluorescence measured in 2xYT medium, which were set as 1.0

To test the influence of different media on the response of the pSenSox‐based NADPH biosensor, the biotransformation of MAA to MHB, catalyzed by the NADPH‐dependent *Lb*Adh, was performed in three complex media (TB, 2xTY, or LB) and in a defined minimal medium (M9) with glucose as carbon source using the experimental setup shown in Figure 1 and as described in the methods section. The experiments with the different media, including control cultures in which MAA was omitted or in which pSenNeg, encoding a defective *Lb*Adh, was used, are shown in Figure 2. MAA itself had a negative influence on growth, even in the absence of *Lb*Adh, and this negative influence was further enhanced in the presence of *Lb*Adh activity, when MAA was reduced to MHB with NADPH as reductant. Regarding the response of the SoxRS‐based NADPH biosensor, the experiments shown in Figure 2 confirmed that expression of the *eyfp* gene is dependent on the biotransformation of MAA to MHB by the *Lb*Adh. In the absence of either MAA or *Lb*Adh activity, eYFP synthesis was not induced.

**Figure A1:**
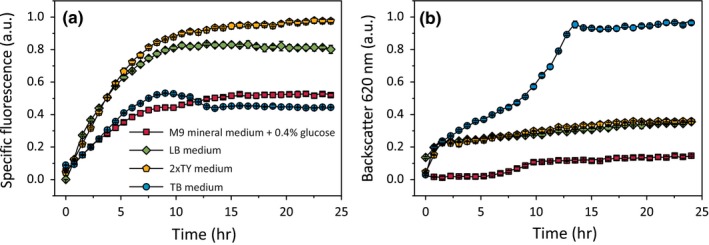
Influence of different media on the NADPH biosensor response (A) and cell density (B) of *Escherichia coli*/pSenSox during biotransformation of 70 mM MAA to MHB. The strain was cultivated in either M9 mineral medium with 0.4% (w/v) glucose, or in LB medium, or in 2xTY medium, or in TB medium. The experimental setup shown in Figure 1 was used for all cultivations in one of the media mentioned above containing 100 μg/ml carbenicillin. Mean values and standard deviations of three independent biological replicates are shown

When comparing the different media, it became obvious that TB allowed by far the best growth, followed by 2xTY and LB medium, in which the cells grew comparably, and M9 glucose medium, in which almost no growth occurred (Figure A1). When comparing the different media with respect to eYFP synthesis, the highest fluorescence after 24 hr was obtained in 2xTY medium and LB medium, whereas it was much lower and comparable for TB and M9‐glucose medium (Figure A1). The almost complete lack of growth in M9‐glucose was due to the biotransformation of MAA to MHB, as growth was observed in the absence of MAA or of *Lb*Adh activity (Figure 2). In this medium, cells have to synthesize all cellular components, in particular amino acids, from glucose, whereas in the other media the presence of yeast extract and tryptone provides amino acids and other cellular components that do not need to be synthesized by the cell but can be imported from the medium. Nevertheless, also in these media the NADPH‐dependent reduction of MAA to MHB had a strong negative effect on growth, presumably due to a lack of NADPH for biosynthetic purposes. An interesting case is TB medium. Although this medium allowed the best growth, the biosensor response was much lower compared to 2xTY or LB and similar to that in M9‐glucose medium. Besides a higher concentration of yeast extract and phosphate buffering, the major difference of TB medium to 2xTY and LB is the presence of glycerol as additional carbon source.

In conclusion, media without a separately added carbohydrate as carbon source, such as 2xTY and LB, led to a higher biosensor response than media containing an added carbohydrate, such as M9‐glucose or TB, which contains 4 ml/L glycerol. This is probably due to a higher NADPH availability by carbohydrate catabolism. M9 glucose medium can in principle be used to monitor the SoxR‐based NADPH biosensor response, which can be necessary or advantageous for experiments in which components of yeast extract or tryptone are disturbing. Overall, the strongest biosensor signal was observed in 2xTY medium, which was therefore chosen for the following experiments.

### 2.2 Influence of redox‐cycling drugs on the NADPH biosensor response

**Figure 3:**
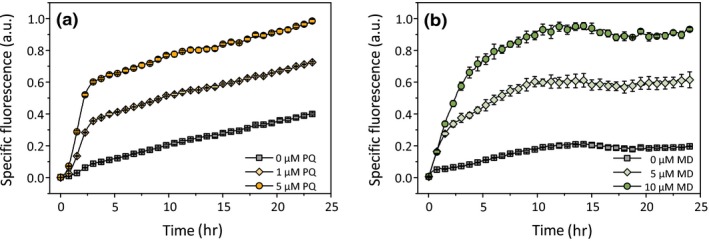
Influence of different concentrations of the redox‐cycling drugs paraquat (PQ) and menadione (MD) on the NADPH biosensor response using *Escherichia coli*/pSenSox. The changes in fluorescence were followed after the addition of either PQ (a) or MD (b) over a time period of 24 hr. The experimental setup shown in Figure 1 was used for all cultivations in 2xTY medium containing 100 μg/ml carbenicillin. No MAA was added in these experiments. Mean values and standard deviations of three independent biological replicates are shown

Paraquat (1,1′‐dimethyl‐4,4′‐bipyridinium dichloride) and menadione (2‐methyl‐1,4‐naphthoquinone) have been reported to induce the *soxRS* regulon in *E. coli* (Greenberg et al., 1990; Seo et al., 2015; Wu & Weiss, 1991). We therefore monitored the response of *E. coli* BL21(DE3)/pSenSox to different paraquat concentrations (0, 1, 5 µM) and different menadione concentrations (0, 5, 10 µM) using the experimental setup shown in Figure 1, except that paraquat and menadione were added instead of MAA. At the concentrations used, both compounds had only minor effects on growth (data not shown), but clearly triggered a concentration‐dependent activation of the SoxRS‐based biosensor response (Figure 3). These results confirm the strong responsiveness of the SoxRS system to paraquat and menadione. Higher concentrations of paraquat (0.01, 0.1, 1, 5, 10 mM) and menadione (15, 20, 25, 50 µM) were tested, but did not lead to further increased fluorescence (data not shown). Addition of up to 5 mM H_2_O_2_ did not elicit a SoxR response (data not shown).

Paraquat and menadione are redox‐cycling drugs, which mediate the transfer of electrons from NADPH to oxygen, leading to the continuous generation of superoxide (Kappus & Sies, 1981). Several possibilities exist how redox‐cycling drugs activate the SoxRS response: (a) the superoxide radical has been shown to directly oxidize the [2Fe‐2S] cluster of SoxR (Fujikawa et al., 2012), thus forming active SoxR; (b) the redox‐cycling drug might directly interact with reduced SoxR and oxidize it, leading to active SoxR (Gu & Imlay, 2011); (c) the reduction of the redox‐cycling agent by NADPH might lead to a decreased NADPH/NADP^+^ ratio, thereby interfering with the NADPH‐dependent reduction of SoxR and causing an increased level of oxidized, active SoxR; (d) the redox‐cycling drugs might be directly reduced by the SoxR‐reducing system(s) of the cell, thereby inhibiting SoxR reduction and causing increased levels of oxidized active SoxR. It is possible that several of these mechanisms contribute to the activation of SoxR by paraquat and menadione.

### 2.3 Influence of *rseC* and *rsxABCDGE* deletion on the NADPH biosensor response

By screening an *E. coli*mutant library, mutations in the *rseC*gene and in the* rsxABCDGE* operon were found to cause constitutive expression of a P*_soxS_*‐*lacZ*reporter gene in a SoxR‐dependent manner (Koo et al., 2003). Further studies led to the conclusion that the membrane‐integral RsxABCDGE complex and the membrane protein RseC constitute a SoxR‐reducing system (Koo et al., 2003). The statement that purified RsxC exhibits NADPH‐dependent cytochrome *c* reduction activity (Koo et al., 2003) suggests that NADPH serves as electron donor of the Rsx complex. To test the influence of this reducing system on the NADPH biosensor response, we constructed deletion mutants of *E. coli*BL21(DE3) lacking either *rseC* (Δ*rseC*), or *rsxABCDGE* (Δ*rsx*), or all of these genes (Δ*rseC*Δ*rsx*).

**Figure 4:**
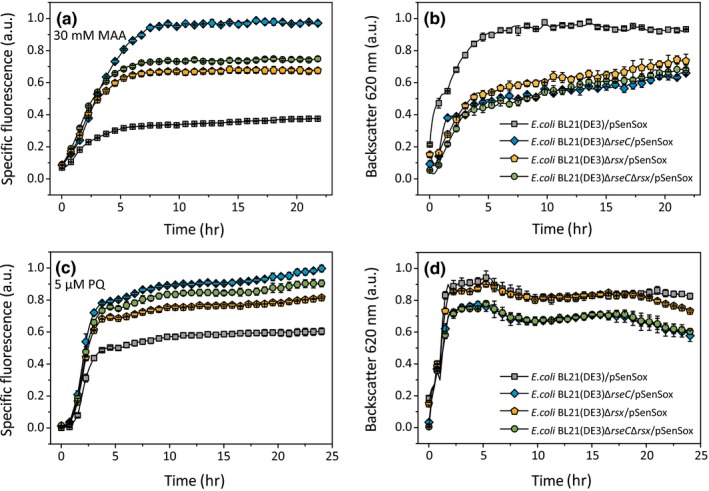
Comparison of the NADPH biosensor response (a, c) and cell density (b, d) of *Escherichia coli*BL21(DE3) and its Δ*rseC*, Δ*rsx*, and Δ*rseC*Δ*rsx* mutants during biotransformation of 30 mM MAA to MHB (panels a and b) using the standard experimental setup shown in Figure 1, and in the presence of 5 µM paraquat instead of MAA (panels c and d). Mean values and standard deviations of three independent biological replicates are shown

**Figure A2:**
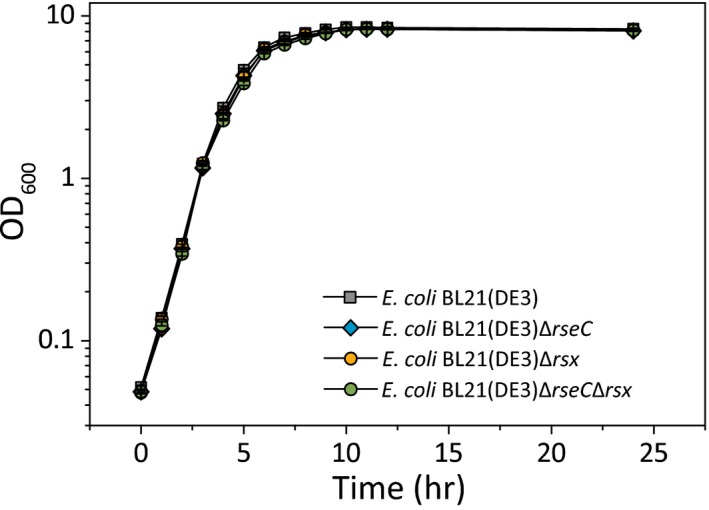
Growth behavior of the mutants *Escherichia coli* Δ*rseC*, *E. coli* Δ*rsx,* and *E. coli* Δ*rse*CΔ*rsx* in comparison to the parental strain *E. coli* BL21(DE3). The strains were cultivated in 50 ml 2xTY medium in 500 ml shake flasks at 30°C and 130 rpm and growth was monitored as OD_600_. Mean values and standard deviations of three independent biological replicates are shown

When monitoring the growth behavior of the deletion mutants and the parental strain in shake flask experiments with 2xTY medium, all strains exhibited the same growth behavior, showing that under these conditions RseC and the Rsx complex are dispensable (Figure A2). The influence of Δ*rseC*, Δ*rsx*, and Δ*rseC*Δ*rsx* mutations on the NADPH biosensor signal was analyzed according to the standard experimental setup shown in Figure 1 with 30 mM MAA as substrate for the NADPH‐dependent *Lb*Adh. As shown in Figure 4, all deletion mutants showed an increased fluorescence signal compared to the parental strain, with the strongest response in the Δ*rseC*mutant, followed by the Δ*rseC*Δ*rsx*mutant and the Δ*rsx* mutant.

**Figure A3:**
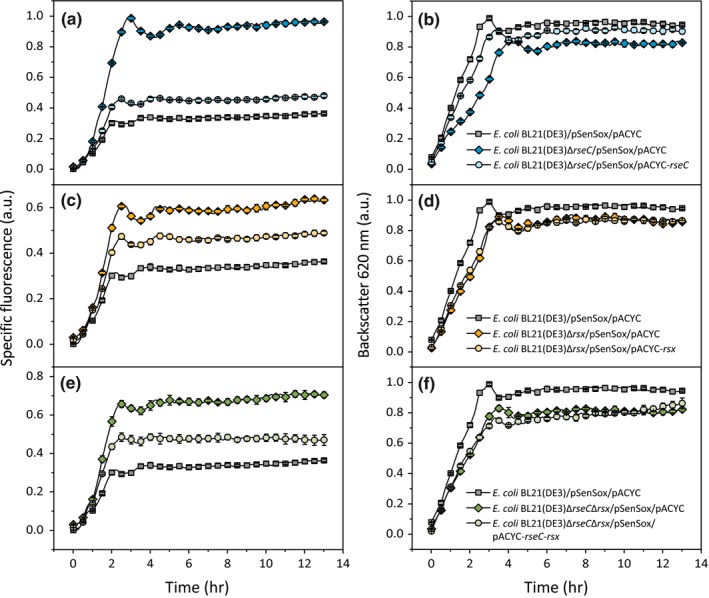
Complementation studies of the Δ*rseC*, Δ*rsx*, and Δ*rseC*Δ*rsx* mutants carrying pSenSox using plasmids pACYC‐*rseC*, pACYC‐*rsx*, and pACYC‐*rseC*‐*rsx*. For comparison, the parent strain carrying pSenSox and pACYCDuet‐1 was used. The experiment was performed according to the standard setup shown in Figure 1 with 30 mM MAA. IPTG was not added to the cultures as it had a negative effect on growth. Mean values and standard deviations of three independent biological replicates are shown

To confirm that the observed effects were due to the gene deletions, plasmids pACYC‐*rseC*, pACYC‐*rsx*, and pACYC‐*rseC*‐*rsx* were constructed and transferred into the corresponding deletion mutants. The parent vector pACYCDuet‐1 served as control. Basal expression of *rseC* and/or *rsxABCDGE* in the respective deletion mutants without addition of IPTG resulted in decreased biosensor signals compared to the ones obtained in the mutants carrying the control plasmid pACYCDuet‐1, but the response was still higher than in the parental strain (Figure A3). Although complementation was only partial, which could be due to an inadequate expression level of the plasmid‐encoded genes, it confirmed that the *rseC* and *rsx* deletions were responsible for the increased biosensor response.

The results described above are in agreement with previous data showing a function of RsxABCDGE and RseC in SoxR reduction (Koo et al., 2003). An interesting observation made by Koo and coworkers and by us was that the deletion of both *rseC* and the *rsx* cluster had no additive effect and expression of the reporter gene was even somewhat lower in the Δ*rseC*Δ*rsx* mutant than in the Δ*rseC* single mutant. This indicates that the Rsx complex and RseC do not function independently to reduce SoxR, but work together, as proposed previously (Koo et al., 2003). The RsxABCDGE complex belongs to the family of Rnf complexes, enzymes that drive the endergonic reduction of ferredoxin (*E*_0_′ = −420 mV) with NAD(P)H (*E*_0_′ = −320 mV) by the proton‐ or sodium‐motive force via import of H^+^ or Na^+^, or, in the reverse reaction, the exergonic reduction of NAD(P)^+^ with reduced ferredoxin coupled to the export of H^+^ or Na^+^ (Biegel, Schmidt, Gonzalez, & Müller, 2011). The redox potential of SoxR in its DNA‐free and its DNA‐bound state was reported to be −293 and −320 mV (Kobayashi, Fujikawa, & Kozawa, 2015), respectively, that is, in the same range as the one of NAD(P)H. Consumption of proton‐ or sodium‐motive force via the Rsx complex to drive reduction of SoxR by NADPH allows the cell to keep most SoxR in the reduced state in the absence of inducing conditions. In fact, it was reported that in wild‐type cells overproducing SoxR almost all of the protein was present in the reduced state, but in *rsxC* and *rseC* mutants only about 60% and 56% (Koo et al., 2003). The final steps of electron transfer to SoxR are unknown at present. In the Rnf complexes, RnfB is suggested as electron donor for ferredoxin (Biegel et al., 2011). Therefore, the homologous RsxB protein could serve to reduce SoxR, or alternatively, RseC might transfer electrons from RsxB to SoxR, as a conserved cysteine motif in the N‐terminal region of RseC could be part of an iron‐sulfur cluster. Further studies are required to solve this issue. The observation that in *rsxC*and *rseC* mutants still about 60% and 56% of SoxR was in the reduced state (Koo et al., 2003) suggests that further enzymes for SoxR reduction exist, which need to be identified.

We also tested the response of the *rsx*and *rseC* mutants in the presence of 5 µM paraquat instead of MAA. Although the parental strain already showed a strong response to paraquat, that of the mutants was still further increased. Again, the Δ*rseC*mutant showed the highest specific fluorescence followed by the Δ*rseC*Δ*rsx*mutant and the Δ*rsxABCDGE* mutant (Figure 4c,d). The observation that paraquat and *rsx* and/or *rseC* deletion showed an additive effect on the biosensor response confirms that a fraction of SoxR must still be in the reduced state in the mutants and suggests that paraquat‐based activation of SoxR is not due to interference with SoxR reduction by the Rsx/RseC system.

### 2.4 Influence of the transhydrogenase deletions *ΔpntAB*, *ΔsthA*, and *ΔsthAΔpntAB* on the NADPH biosensor response

Transhydrogenases catalyze the reversible interconversion of NADH and NADPH. *E. coli*possesses two transhydrogenases, the membrane‐bound, proton‐translocating transhydrogenase PntAB and the soluble, energy‐independent transhydrogenase SthA (also called UdhA; Sauer, Canonaco, Heri, Perrenoud, & Fischer, 2004). Due to the relevance of transhydrogenases in the regulation of cellular NADPH levels, we studied the influence of these enzymes on the NADPH biosensor response by constructing deletion mutants of *E. coli*BL21(DE3) lacking either *sthA*, or *pntAB*, or both.

**Figure A4:**
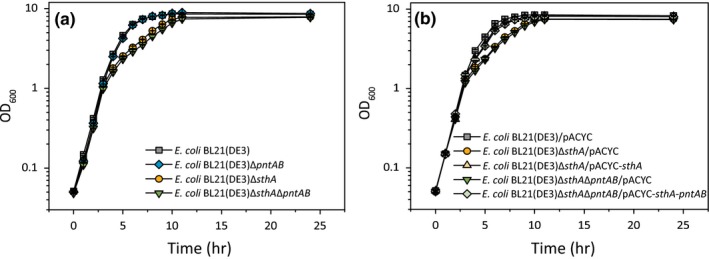
Growth (OD_600_) of the transhydrogenase mutants Δ*pntAB,* Δ*sthA,*and Δ*sthA*Δ*pntAB* compared to the parental strain *Escherichia coli* BL21(DE3) (A) and complementation of the Δ*sthA*and Δ*sthA*Δ*pntAB* strains (B). The strains were cultivated at 30°C and 130 rpm in 500 ml shake flasks with 50 ml of 2xTY medium. Complementation studies were performed in the presence of 0.1 mM IPTG and 34 μg/ml chloramphenicol. Mean values and standard deviations of three independent biological replicates are shown

**Figure A5:**
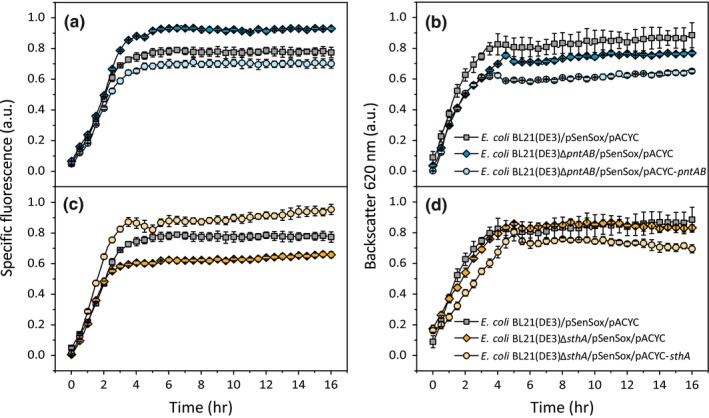
Complementation studies of the Δ*pntAB* and Δ*sthA* mutants carrying plasmid pSenSox with plasmids pACYC‐*pntAB* or pACYC‐*sthA*. For comparison, the parent strain and the mutants carrying pSenSox and pACYCDuet‐1 (pACYC) were used. The experiment was performed according to the standard setup shown in Figure 1 with 30 mM MAA. Mean values and standard deviations of three independent biological replicates are shown

The growth behavior of the transhydrogenase mutants was tested in shake flask experiments using 2xTY medium. Whereas the Δ*pntAB*mutant grew like the parental strain, the Δ*sthA*mutant and the Δ*sthA*Δ*pntAB* double mutant showed a growth defect that became apparent during the exponential growth phase (Figure A4). Presumably, an excess of NADPH is formed in this growth phase, which cannot be readily diminished in the absence of SthA. The defect could be largely abolished by plasmid‐based expression of *sthA* (Figure A5), confirming that it was caused by the *sthA* deletion.

**Figure 5:**
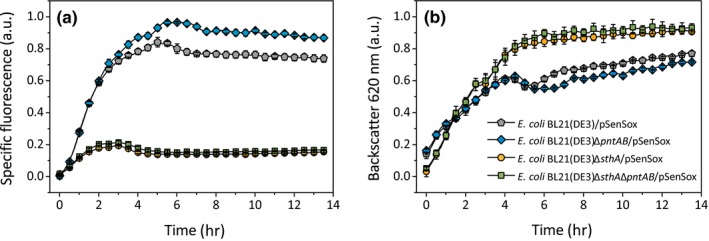
Comparison of the NADPH biosensor response (a) and cell density (b) of *Escherichia coli*/pSenSox and its Δ*pntAB*, Δ*sthA*, and Δ*sthA*Δ*pntAB* mutants during biotransformation of MAA (30 mM) to MHB using the standard experimental setup shown in Figure 1. Mean values and standard deviations of three independent biological replicates are shown

The influence of SthA and PntAB on the NADPH biosensor signal was analyzed according to the standard experimental setup shown in Figure 1 with 30 mM MAA as substrate for the NADPH‐dependent *Lb*Adh. Whereas the Δ*pntAB* mutant displayed a slightly increased biosensor signal, it was decreased by more than 60% in the Δ*sthA* mutant and also in the Δ*sthA*Δ*pntAB* double mutant (Figure 5). The latter result showed that the *sthA* deletion was dominant over the *pntAB* deletion.

To confirm that the observed effects were due to the gene deletions, plasmids pACYC‐*pntAB* and pACYC‐*sthA* were constructed and transferred into the corresponding deletion mutants. The parent vector pACYCDuet‐1 served as control. Plasmid‐based expression of *pntAB* in the Δ*pntAB* mutant completely prevented the increase in the biosensor signal and even reduced it to a small extent. Vice versa, plasmid‐based expression of *sthA* in the Δ*sthA*mutant completely prevented the decrease in the sensor response and even increased it (Figure A5). The presence of a second plasmid reduced the differences in the biosensor signal between the Δ*sthA*mutant and the parent strain, which might be due to a negative effect on the copy number of pSenSox and thus *Lb*Adh activity.

The results obtained with the Δ*pntAB*mutant indicate that, under the conditions used, PntAB catalyzes NADP^+^ reduction by NADH, leading to an increased NADPH availability. Absence of *pntAB* therefore results in a lowered NADPH availability and thus an increased biosensor signal. This conclusion is in agreement with previous studies showing that PntAB is involved in NADPH formation in *E. coli*(Sauer et al., 2004) and that overexpression of *pntAB* enhanced conversion of acetophenone to (*R*)‐phenylethanol by the NADPH‐dependent alcohol dehydrogenase of *Lactobacillus kefir* (Weckbecker & Hummel, 2004) and improved the biosynthesis of 3‐hydroxypropionic acid from its precursor malonyl‐CoA by an NADPH‐dependent malonyl‐CoA reductase (Rathnasingh et al., 2012). Moreover, heterologous overexpression of the *E. coli pntAB* genes in *Corynebacterium glutamicum* was shown to enhance production of l‐lysine, whose biosynthesis is strongly NADPH‐dependent (Kabus, Georgi, Wendisch, & Bott, 2007).

In contrast to PntAB, our results obtained with the Δ*sthA* mutant indicate that under the conditions employed SthA catalyzes NADPH oxidation. Absence of *sthA* thus results in an increased NADPH availability, which is reflected by a decreased biosensor signal. These data are in agreement with previous studies showing that SthA is required under conditions leading to excess NADPH formation (Sauer et al., 2004). The observation that *pntAB* deletion in the Δ*sthA* mutant did not reverse the decrease in the biosensor signal suggests that SthA activity is much higher than PntAB activity, which can be expected based on the fact that PntAB catalyzes a reaction coupled to proton transfer across the membrane. In conclusion, our data confirm that PntAB and SthA play important and opposite functions for NADPH availability in *E. coli*.

### 2.5 Concluding remarks

In this study, we employed the NADPH biosensor pSenSox to study various processes expected to influence the NADPH availability in *E. coli*. We could confirm that the lack of *rsxABCDGE* and *rseC* activated the pSenSox‐based response. Paraquat and the Δ*rsx*/Δr*seC* deletion had an additive effect on the sensor response, indicating that further SoxR‐reducing systems exist besides Rsx/RseC and that the paraquat‐induced response is presumably not due to interference of paraquat with SoxR reduction by Rsx/RseC. The transhydrogenases PntAB and SthA had opposite effects on the biosensor response, in agreement with PntAB being involved in NADP^+^ reduction and SthA catalyzing NADPH oxidation. In conclusion, the pSenSox‐based NADPH biosensor is a useful tool not only for HT‐screening of the activity of NADPH‐dependent alcohol dehydrogenases (Siedler et al., 2014), but also for analyzing conditions and proteins influencing NADPH availability or SoxR reduction. Recently, another NADPH biosensor was described, which is based on the specific oxygen‐independent amplification of the intrinsic fluorescence of NADPH by the mBFP protein (Hwang, Choi, Han, & Kim, 2012). The mBFP protein was shown to be well suited to study the dynamics of intracellular NADPH availability with a resolution of seconds and to allow the quantitation of NADPH (Goldbeck, Eck, & Seibold, 2018). This is not possible with the pSenSox NADPH biosensor, which requires transcription, translation, and oxygen‐dependent maturation of eYFP. However, pSenSox allows to preserve changed NADPH levels as a stable fluorescence signal, which is a prerequisite, for example, for FACS‐based screening of mutant libraries of NADPH‐dependent enzymes, and it allows to specifically analyze the effects of enzymes that are involved in SoxR reduction or oxidation, which is presumably not possible with the mBFP sensor. Thus, mBFP and pSenSox represent two different types of NADPH biosensors, which both have their specific advantages and application fields.

## 3 MATERIALS AND METHODS

### 3.1 Bacterial strains, plasmids, and growth conditions

All strains and plasmids used in this work are listed in Table 1. *E. coli* BL21(DE3) (Invitrogen, Karlsruhe, Germany) and its derivatives were used for all studies with the NADPH biosensor. *E. coli* NEB5α was employed for cloning purposes. Transformation of *E. coli* cells was performed as described (Hanahan, 1983). Cells were cultivated at 30°C or 37°C in lysogeny broth (LB; Miller, 1972), in 2xTY (16 g/L tryptone, 10 g/L yeast extract, 5 g/L sodium chloride), in M9 mineral medium (33.7 mM Na_2_HPO_4_ × 2H_2_O, 22.0 mM KH_2_PO_4_, 8.55 mM NaCl, 9.35 mM NH_4_Cl, 1 mM MgSO_4_ × 7H_2_O, 0.3 mM CaCl_2_, 1 µg/ml biotin, 1 µg/ml thiamin, trace elements) supplemented with 0.4% (w/v) glucose (Sambrook & Russell, 2001), or in terrific broth (TB) medium (12 g/L tryptone, 24 g/L yeast extract, 4 ml glycerol, 12.54 g/L K_2_HPO_4_, 2.31 g/L KH_2_PO_4_; pH 7.0). Plasmids were selected by adding antibiotics to the medium to a final concentration of 100 µg/ml carbenicillin, 34 µg/ml chloramphenicol, or 50 µg/ml kanamycin.

**Table 1:** Bacterial strains and plasmids used in this study

Strain or plasmid	Relevant characteristics	Source or reference
*Escherichia coli*		
NEB5α	*fhuA2 Δ(argF‐lacZ)U169 phoA* *glnV44 Φ80Δ (lacZ)M15 gyrA96 recA1 relA1 endA1 thi‐1 hsdR17*; strain used for general cloning procedures	New England Biolabs
BL21(DE3)	F‐ *ompT* *hsdS*_B_ (r_B_−, m_B_−) *gal dcm* (DE3); parent strain used in this study	Studier and Moffatt (1986)
BL21(DE3)Δ*rseC*	Derivative of BL21(DE3) with an in‐frame deletion of *rseC*, locus‐tag ECD_02464	This study
BL21(DE3)Δ*rsx*	Derivative of BL21(DE3) with an in‐frame deletion of *rsxABCDGE,*locus‐tag ECD_01597‐ECD_01602	This study
BL21(DE3)Δ*rseC*Δ*rsx*	Derivative of BL21(DE3) with an in‐frame deletion of *rseC* and *rsxABCDGE,*locus‐tags *ECD_02464,*and *ECD_01597 ‐ ECD_01602*	This study
BL21(DE3)Δ*sthA*	Derivative of BL21(DE3) with an in‐frame deletion of Δ*sthA,*locus‐tag ECD_03847	This study
BL21(DE3)Δp*ntAB*	Derivative of BL21(DE3) with an in‐frame deletion of Δ*pntAB,*locus‐tags ECD_01571 and ECD_01572	This study
BL21(DE3)Δ*sthA*Δ*pntAB*	Derivative of BL21(DE3) with an in‐frame deletion of Δ*sthA* and Δ*pntAB*), locus‐tags ECD_03847, ECD_01571 and ECD_01572	This study
Plasmids		
pSenSox	Amp^R^; pBtac‐*Lbadh* derivative containing the *soxRS*‐based NADPH biosensor and the *Lactobacillus brevis adh* gene under control of the *tac* promoter	Siedler et al. (2014)
pSenNeg	Amp^R^; pSenSox derivative with an incomplete Lb*adh* gene preventing synthesis of an active *Lb*Adh	Siedler et al. (2014)
pSIJ8	Amp^R^; lambda Red‐mediated gene replacement vector expressing lambda Red recombinase and flippase recombinase genes (pKD46, rhaRS‐prha‐FLP, amp)	Addgene; Jensen et al. (2015)
pKD4	Kan^R^; template plasmid for FRT‐flanked *kan* cassette needed for lambda Red‐mediated gene replacement	Addgene; Datsenko and Wanner (2000)
pACYCDuet‐1	Cm^R^; vector for the coexpression of two target genes, each under the control of a separate T7 promoter and associated ribosomal binding site (p15A *oriV_ec_*, 2(P*_T7_*), *lacI*	Merck Millipore
pACYC‐*rseC*	Cm^R^; pACYCDuet‐1‐derivative for expression of *rseC* under the control of the T7 promoter	This study
pACYC‐*rsx*	Cm^R^; pACYCDuet‐1‐derivative for expression of *rsxABCDGE* under the control of the T7 promoter	This study
pACYC‐rseC‐rsx	Cm^R^; pACYCDuet‐1‐derivative for expression of *rseC*and* rsxABCDGE* under the control of the T7 promoter	This study
pACYC‐*sthA*	Cm^R^; pACYCDuet‐1‐derivative for expression of *sthA* under the control of the T7 promoter	This study
pACYC‐*pntAB*	Cm^R^; pACYCDuet‐1‐derivative for expression of *pntAB* under the control of the T7 promoter	This study
pACYC‐*sthA*‐*pntAB*	Cm^R^; pACYCDuet‐1‐derivative for expression of *sthA*and* pntAB* under the control of the T7 promoter	This study

### 3.2 Recombinant DNA work and construction of deletion mutants

Standard methods such as PCR and DNA restriction enzyme digestion were carried out according to established protocols (Sambrook & Russell, 2001). Oligonucleotides were synthesized by Eurofins Genomics (Ebersberg, Germany) and are listed in Table A. Construction of pACYC‐*rseC*, pACYC‐*rsx*, pACYC‐*rseC*‐*rsx*, pACYC‐*sthA*, and pACYC‐*pntAB*was performed by Gibson assembly (Gibson et al., 2009). All plasmids were sequenced by Eurofins Genomics, Ebersberg, Germany. The construction of *E. coli* deletion mutants was performed with the lambda Red recombinase method (Datsenko & Wanner, 2000) using a recent protocol (Jensen, Lennen, Herrgard, & Nielsen, 2015). For the markerless deletion of genes, the arabinose‐inducible lambda Red recombineering genes (*exo*, *bet*, and *gam*) and the rhamnose‐inducible flippase (FLP) recombinase were introduced into *E. coli* BL21(DE3) using the temperature‐sensitive plasmid pSIJ8 (Jensen et al., 2015). The FRT‐flanked kanamycin cassette, which was integrated into the *E. coli* genome at the locus of the gene to be deleted, was encoded by plasmid pKD4. For amplification of the FRT‐flanked kanamycin cassette of pKD4, oligonucleotides that carried homology regions to the up‐ and downstream regions of the genes to be deleted were used. All gene deletions were verified by colony PCR using DreamTaq Master Mix 2X (Thermo Scientific, Schwerte, Germany) and the oligonucleotides listed in Table A.

### 3.3 Monitoring the NADPH biosensor response

The NADPH biosensor response during the whole‐cell biotransformation of MAA to MHB by the strictly NADPH‐dependent *Lb*Adh was measured as described (Siedler et al., 2014). The specific fluorescence is defined as the ratio of fluorescence and backscatter. The biotransformations were performed with *E. coli* BL21(DE3) and its derivatives, such as deletion mutants lacking the genes coding for the SoxR‐reducing system or transhydrogenases, and mutant strains carrying expression plasmids for the deleted genes. The *E. coli* BL21(DE3) cells were transformed either with plasmid pSenSox carrying the SoxR‐based NADPH biosensor and an intact *Lb*Adh gene or with plasmid pSenNeg, in which a part of the *Lbadh* gene was deleted to prevent synthesis of an active *Lb*Adh (Siedler et al., 2014).

The experimental setup used for the biotransformation experiments is shown in Figure 1. Pre‐cultures of three biological replicates of the desired strains were incubated overnight at 37°C and 130 rpm in 5 ml medium containing the appropriate antibiotic(s) as selection marker(s). These pre‐cultures were used for the inoculation of the main cultures to an optical density at 600 nm (OD_600_) of 0.05. The main cultures were grown in 50 ml medium in the presence of the appropriate antibiotics at 37°C and 130 rpm using 500 ml shake flasks. For complementation experiments, the cells were incubated at 30°C. Basal expression of *Lbadh* by the non‐induced *tac* promoter allowed for sufficient *Lb*Adh activity in the biotransformation experiments (Siedler et al., 2014). When a higher *Lb*Adh activity was required, IPTG was added to a final concentration of 0.1 mM when the cultures had reached an OD_600_ between 0.6–0.8. To ensure that enough biomass is present for the biotransformation, the cultures were further incubated for at least 5 hr, until an OD_600_ of 5 or higher was reached. Then the cells were harvested by centrifugation (4°C, 4,713 *g* and 15 min) and resuspended in fresh medium supplemented with the corresponding antibiotic to a final OD_600_ of 5. About 800 µl of these suspensions were transferred into 48‐well microtiter Flowerplates (m2p‐labs, Baesweiler, Germany). When analyzing the biosensor response under conditions of reductive biotransformation, 100 µl MAA dissolved in ddH_2_O at the desired concentration was added to the 800 µl cultures or 100 µl ddH_2_O as negative control. To study the effect of redox‐cycling drugs and hydrogen peroxide on the biosensor response, the following additions were made to the 800 µl cultures in the Flowerplates: (a) 100 µl paraquat (1,1′‐dimethyl‐4,4′‐bipyridinium dichloride; Sigma‐Aldrich) dissolved in ddH_2_O to final concentrations of 1 and 5 µM; (b) 100 µl menadione (2‐methyl‐1,4‐naphthoquinone; Sigma‐Aldrich) dissolved in DMSO to final concentrations of 5 and 10 µM; (c) 100 µl H_2_O_2_ to final concentrations of 0.05 or 5 mM. For the negative controls, 100 µl ddH_2_O or 100 µl DMSO was added.

After the desired additions, the Flowerplates were incubated in a BioLector microcultivation system (m2p‐laps; Baesweiler, Germany) at 30°C and 1,200 rpm (shaking diameter 3 mm), which allows online monitoring of cell density (as backscattered light at 620 nm) and of eYFP fluorescence (excitation wavelength 485 nm, emission wavelength of 520 nm). For the different experiments, the values were normalized to the maximal backscatter and the maximal specific fluorescence observed, which were set as 1.0.

## CONFLICT OF INTEREST

The authors declare no conflict of interest.

## AUTHORS CONTRIBUTION

A.S., M.Ba., and M.Bo. designed research; A.S. performed all experiments; A.S., M.Ba., and M.Bo. analyzed the data; A.S. prepared the figures; A.S., M.Ba., and M.Bo. wrote the manuscript.

## ETHICAL APPROVAL

None required.

## Data Availability

All data are included in the article.

## Appendix A

**Table A1:** Oligonucleotides used in this study

Oligonucleotide	Sequence (5′→3′) and properties
Oligonucleotides used for the construction of deletion mutants with the lambda recombination system with 5′‐parts corresponding to the upstream or downstream region of the gene(s) to be deleted (shown in bold) and 3′‐parts corresponding to the FRT‐flanked kanamycin cassette of plasmid pKD4.	
rseC fw	**GAAATGTTCATACCGTATGGATTGTGGTATCTGGAAACGTCCTCGCATTTGTTATGCAAAATGCAACAAAGCCAGTGAAA**GTGTAGGCTGGAGCTGCTTC
rseC rv	**GCCGAAATCACCATTGTCGGTGAACTGCCGCCGCAAACGGCGAAACGCATTGCCGAGAATATTAAGTTCGGGGCAGCGCA** ATGGGAATTAGCCATGGTCC
rsxABCDGE fw	**GTAATAACCTTACAGTTAACCTGTTGTCGCCTGCTCTGGATTAACGGATAATAGGCGGCTTTTTTATTTCAGGCCGAAAA**GTGTAGGCTGGAGCTGCTTC
rsxABCDGE rev	**TCAAAAGGCGAACTGAAATTAAGCTCGGTGGTGGGATGAGGATTGTTCTCACGCAGGCGAGTGAGGATCTCCAGGCGTTT**ATGGGAATTAGCCATGGTCC
pntAB fw	**ATTTAGCTCGTACATGAGCAGCTTGTGTGGCTCCTGACACAGGCAAACCATCATCAATAAAACCGATGGAAGGGAATATC**GTGTAGGCTGGAGCTGCTTC
pntAB rv	**AAACAGCGGGAGGTCAAAGCATCGCCGTTCTGTTAAGCGATCTCAATAAAGAGTGACGGCCTCAGCAGAGGCCGTCAGGG**ATGGGAATTAGCCATGGTCC
sthA fw	**TGACGGGGATCAATTGGCTTACCCGCGATAAAATGTTACCATTCTGTTGCTTTTATGTATAAGAACAGGTAAGCCCTACC**GTGTAGGCTGGAGCTGCTTC
sthA rv	**CTGGCTCACCGCTGCGCAGCCGCTATGAGCAGCTGGCAGAGGCCATCCGCGCAAGAATGGATGGCCATTTCGATAAAGTT**ATGGGAATTAGCCATGGTCC
Oligonucleotides used for cloning of genes into pACYCDuet‐1 with overlapping regions required for Gibson assembly shown in bold.	
rseC‐pACYC fw	CTTTAATAAGGAGATATACATGATCAAAGAGTGGGCTACC
rseC‐pACYC rv	CATTATGCGGCCGCATCACTGGCTCGCGTCTTC
rsxABC‐pACYC‐fw	**GGAGATATACATATGGCA**ATGACTGACTACCTGTTAC
rsxABC‐rsxDGE rev	**GAATACCAT**TTAGTCCTCGTTTACAACC
rsxDGE‐rsxABC fw	**GAGGACTAA**ATGGTATTCAGAATAGCTAGC
rsxDGE‐pACYC rev	**GCGTGGCCGGCCGAT**TCAGACATTCCCTGTTTC
Oligonucleotides used for colony‐PCRs and sequencing of plasmids	
Δ*rseC* verification fw	GACGGTCGGCGTTATAGGTC
Δ*rseC* verification rev	GGACGCAGAACCGTCAGTACAAGCG
Δ*rsxABCDGE* verification fw	GTTGGCAACCGCATTGATCG
Δ*rsxABCDGE*verification rv	CCCGGCGCAAATTGAGTACG
Δ*sthA* verification fw	TGCTGCGTGAGGTGAAAGTC
Δ*sthA* verification rv	AGTCCGCAAGTTCCGCAATG
Δ*pntAB* verification fw	GCTCAAATGACCGTCTATGC
Δ*pntAB* verification rv	GATTGCTGGCCTTTGCGCTT
pACYCDuet‐1 MCS_1 sequencing fw	CAGCAGCCATCACCATCATC
pACYCDuet‐1 MCS_2 sequencing fw	CGAAATTAATACGACTCACTATAGG
